# Endoscopic retrieval of an impacted pancreatic duct basket using a cystotomy knife: A case report

**DOI:** 10.1055/a-2864-1488

**Published:** 2026-07-16

**Authors:** Ping-Ping Zhang, Hong-Xin Sun, Yu Zhang, Liang-Hao Hu

**Affiliations:** 1 Department of Gastroenterology 12521 Changhai Hospital, Naval Military Medical UniversityShanghaiChina

A 54-year-old man was admitted to our hospital due to pancreatic duct (PD) stones. The patient had undergone endoscopic retrograde cholangiopancreatography (ERCP) for stone removal at another hospital, and there had been a basket incarceration during the operation. The patient underwent three sessions of extracorporeal shock wave lithotripsy, followed by ERCP. 1​ During the ERCP procedure, a PD stent and fractured remnant of a retrieval basket (four-wire type) were observed at the papillary opening ( Fig. 1 ). An attempt to remove the stent using an snare was unsuccessful. X-ray imaging showed that a basket shadow containing extensive filling defects was visible in the body of the pancreas ( Fig. 2 ).

**Fig. 1:**
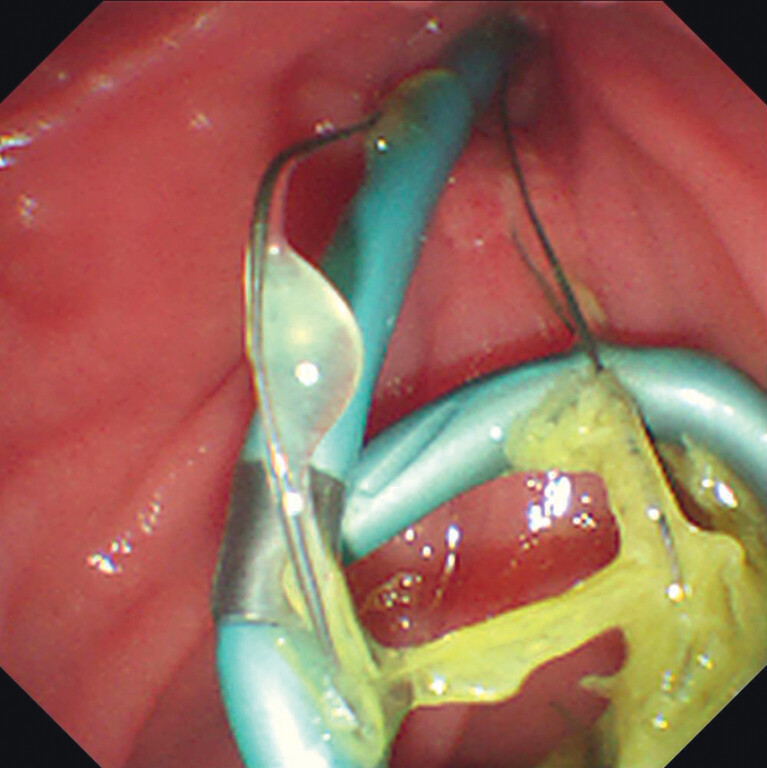
A pancreatic duct stent and fractured remnant of a retrieval basket were observed at the papillary opening.

**Fig. 2:**
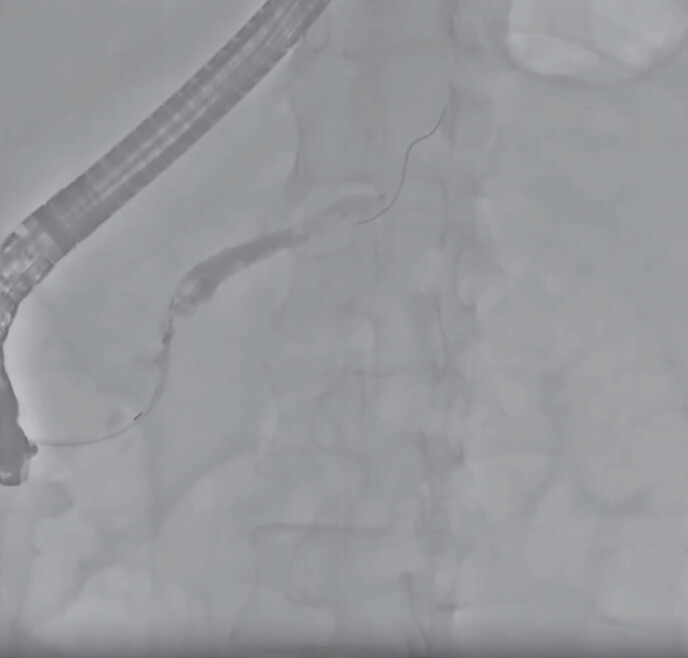
X-ray imaging showed that a basket shadow containing extensive filling defects was visible in the body of the pancreas.

A three-stage dilation balloon catheter (specification: 8-9-10 mm) was used to dilate the PD ( Fig. 3 ), and attempt to drag out the basket was unsuccessful. The choledochoscope was inserted to explore the PD, and a metal mesh basket and numerous white pancreatic stones were revealed in the the PD. The U100 plus EHL lithotripsy fiber was inserted into the surface of the stone through the working tube of an endoscope under direct visualization for electrohydraulic lithotripsy, with a monopulse energy of 160 mJ and a frequency of 120 Hz. After the procedure, the pancreatic stones were broken into small stones ( Fig. 4 ). The choledochoscope was considered that the basket was adhered to the PD tissue. Therefore, a snare was threaded through a 10 F cystotomy knife. The end of the basket was grasped by the snare and then retracted into the cystotomy knife. The knife was advanced to the site of adhesion between the basket and the PD for electrocoagulation and resection, and the mesh basket was successfully separated from the PD wall and retrieved via coagulation and cutting currents ( Fig. 5 , Video 1 ).

**Fig. 3:**
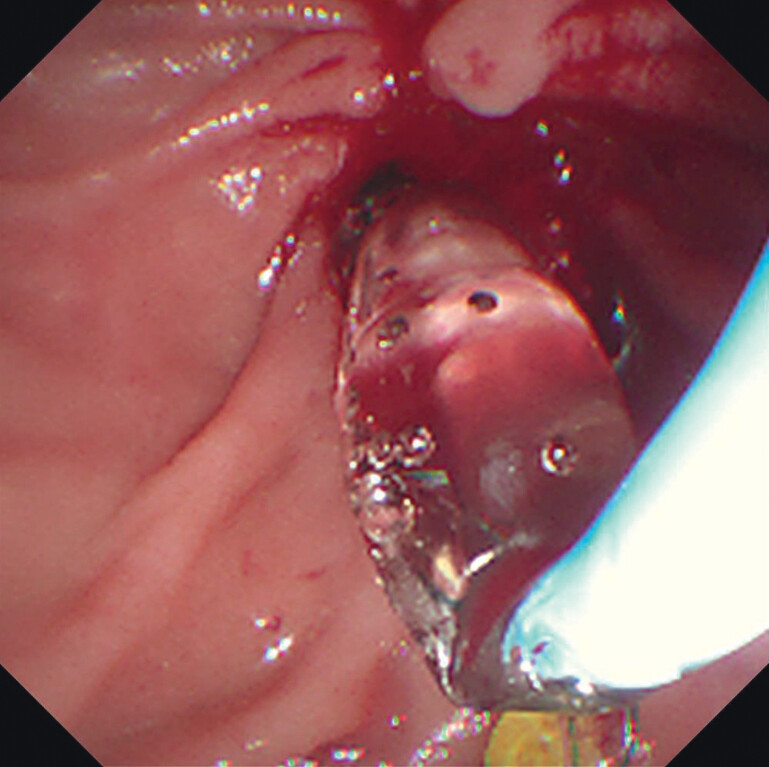
A three-stage dilation balloon catheter was used to dilate the pancreatic duct.

**Fig. 4:**
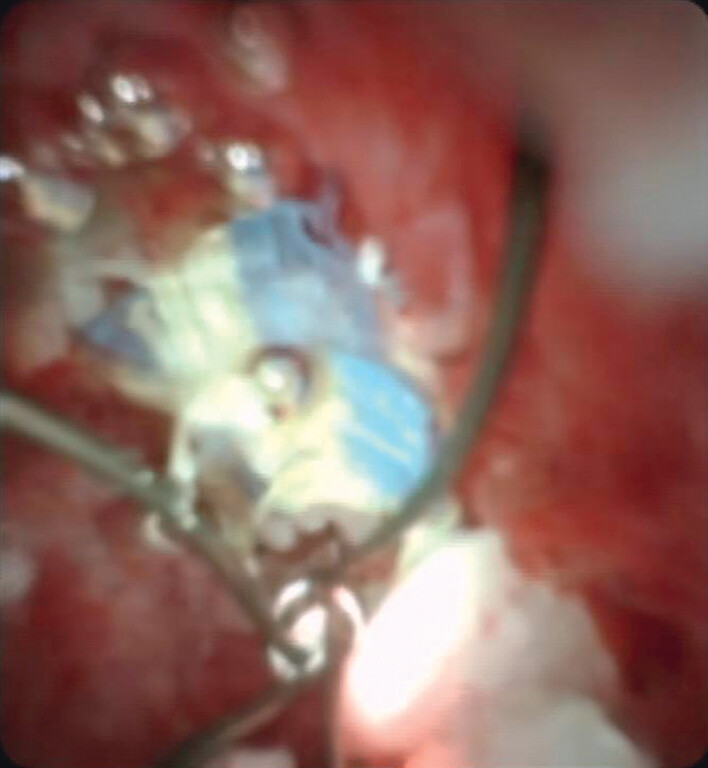
The choledochoscope was introduced via the biopsy channel of the endoscope, and a laser was introduced through the accessory channel and fragmenting the stone.

**Fig. 5:**
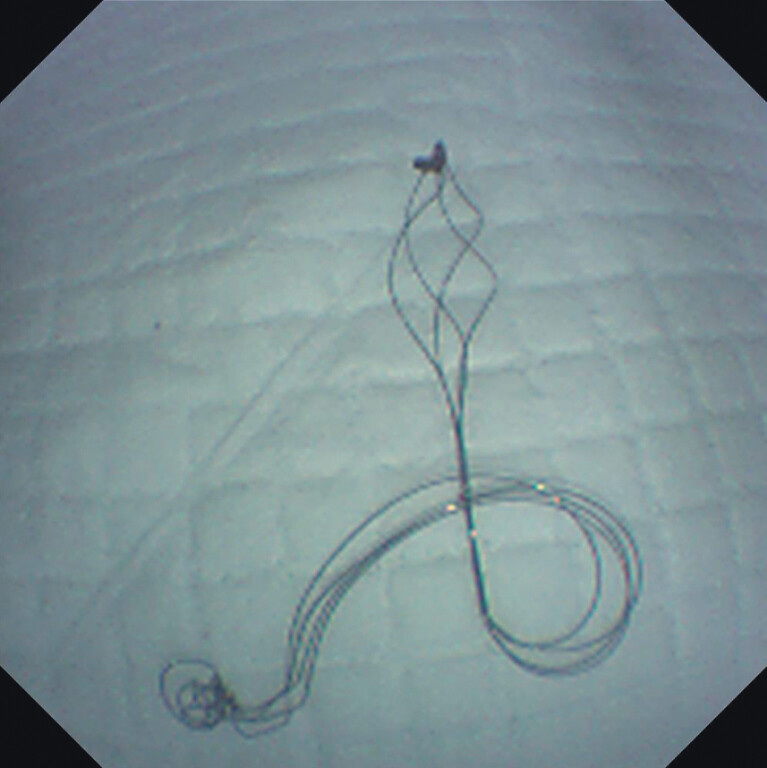
The cystotomy knife was advanced to the site of adhesion between the basket and the pancreatic duct, and the mesh basket was successfully separated from the pancreatic duct wall and retrieved via coagulation and cutting currents.

**Video 1** The PD stent fractured basket removed via EHL lithotripsy, snare coagulation, and cutting.


Endoscopy_UCTN_Code_CPL_1AK_2AI

## List of Abbreviations

ESWL: Extracorporeal shock wave lithotripsy
ERCP: Endoscopic retrograde cholangiopancreatography
